# Buffalo Medical and Surgical Association

**Published:** 1890-08

**Authors:** W. H. Bergtold

**Affiliations:** Secretary


### BUFFALO MEDICAL AND SURGICAL ASSOCIATION.

Reported by W. H. BERGTOLD, M. D., Secretary.

Regular meeting held July 1st, in the Parlors of the Hotel Iroquois, at 8.45 P. M.

The president, Dr. A. A. Hubbell, in the chair. Dr. F. Thoma was proposed for membership. Dr. T. Haven Ross was elected to full membership.

Dr. A. H. Briggs then presented a case of cyanosis in the person of a boy, fourteen years old. The boy had suffered from the various infant maladies with no unusual results, and had always appeared healthy until the age of eight. At this time he had an attack which was difficult at this late date to diagnosticate. He then suffered from acute sharp pain in all of the greater joints, with contractures of the surrounding and contiguous muscles. There appears to have been pyrexia, and the attack is said to have lasted about three weeks.

Toward the end of this sickness the present condition of cyanosis appeared. At the present time the boy is in his most favorable condition, for when very hot weather ensues he suffers from severe pains in the joints, great dyspnea, and excessive heat. Examination reveals a faint first heart sound, action rapid, but no murmurs ; lungs apparently normal.

The skin all over the body is of a bluish hue, the conjunctivæ are cyanotic, the fingers are markedly clubbed, and the nails livid. As said before, when the warm months appear, his condition becomes much worse, the eyes appear greatly enlarged, the lungs seem congested, and give rise to excessive dyspnea ; he experiences great pain in the joints, and the heart is very labored in action.

The mucous membranes then become almost black, and the tongue swells. Has never had any convulsions ; can sleep well, in fact requires more sleep than a boy usually does, and has no difficulty of locomotion.

DISCUSSION.

Dr. W. S. Tremaine.—With a condition of faint first sound, and strong apex, he would be led to suspect obstruction somewhere in the greater vessels ; perhaps from an embolus which might have lodged, and caused a narrowing of a vessel. It might be, it is conceivable, a case of general telangiectasis, though he had never heard of a case.

Dr. P. W. Van Peyma asked why Dr. Briggs concluded it was from pulmonary obstruction. Was it because of his examination or because of his line of reasoning ? Most of these cases of cyanosis are due to pulmonary lesions.

Dr. F. W. Bartlett takes Dr. Tremaine's view of the case, and would locate the obstruction somewhere in the venous system. He did not think it could be due to a neoplasm, because of the long standing of the case.

Dr. Callahan did not think the trouble was now due to an embolus, for it has existed too long.

Dr. Grosvenor did not think it was possible to make an exact diagnosis without a complete history and examination. He asked what had been done for the patient in the line of therapeutics.

Dr. W. H. Bergtold said that cases of cyanosis are due to one of two causes ; either an obstruction to the circulation, preferably in the lungs ; or to a communication between the right and left heart, or between the pulmonary artery and the aorta.

From the previous history of acute pain in the joints with muscular spasm, and a continuation of the same for some time with the supervention of the cyanosis, it is imaginable that the lad had suffered from a general infection of staphylococci ; as, for example, general osteo-myelitis, with secondary infection of the endocardium, giving rise to ulceration and perforation of the inter-auricular or ventricular septum, leaving a permanent opening. He mentioned a case he had seen lately, of an Indian, about forty-five years old, who was excessively cyanotic and who he believes has a patulous foramen ovale, though he had never examined him. This case when quiet is several shades lighter than when exercising, a change, it seems to Dr. Bergtold, to be wholly in accord with the condition theorized to exist; for, with stronger hearts' action, more venous blood would probably be forced into the left heart, via the foramen.

Dr. Tremaine thought the last point well taken, and said that the case was one of great interest. It ought to be thoroughly worked up and published.

Dr. Alvin A. Hubbell then presented a

### NEW TEST FOR DETERMINING THE ABSENCE OR PRESENCE OF EQUILIBRIUM OF THE EXTERNAL OCULAR MUSCLES.

Since so much disturbance of the nervous system is referable to strain of the muscles of the eyes,—the ciliary, as in refractive errors and affections of accommodation, and the extra-ocular, as in want of proper functional balance,—it is desirable that tests for determining the existing states should be made as simple and universal as possible. At the very best, extensive apparatus and special skill seem to be necessary to estimate refractive and accommodative errors, and heretofore the same has been true of anomalies of the external ocular muscles. Recently, however, Maddox, of Edinburgh, has suggested a simple method for determining the latter. I have tried his instrument and found it so satisfactory and easy of application, that I desire to call the attention of the profession further to it.

The instrument consists of a glass rod about one-fourth of an inch in diameter and three-fourths of an inch long, set in a metallic disc or frame-work, so that light cannot pass around it at any point, but only through it.

When held before one eye, viewing a lamp-light in an otherwise darkened room, the blaze appears to be simply a streak of light running at right angles to the axis of the glass rod. The patient should be placed fifteen to twenty feet from the light to be used.

To test the external and internal recti muscles, this instrument is held before one eye with its axis horizontal. The vision of both eyes then being directed toward the light, both the lamp-blaze and the streak of light are seen, and if the action of the muscles is balanced, the latter will pass vertically directly through the former. If the external recti muscles are too weak as compared with the interni, the streak of light will be to one side of the blaze, and to the side corresponding with the eye before which the glass cylinder is placed. The prism, with base toward the temple,— that is, out,— which brings and holds the light-streak through the blaze, measures the amount of deviation inwards of the eyes,— esophoria. On the other hand, if the interni muscles are too weak, the streak will be on the side of the blaze opposite to the eye before which the instrument is placed, and the prism with base in, that is, toward the nose, which brings the streak through the blaze, measures the deviation of the eyes outward,— exophoria. A deviation of three to eight degrees inward, or of eight to fifteen degrees outward, is sufficient to cause distress.

To test the state of the superior and inferior recti muscles, the glass cylinder is placed with its axis vertical. Then, if there is a correct balance, the horizontal streak of light will pass directly through the blaze. If it is below the blaze, the eye before which it is placed has a deviation upward or hyperphoria, and the prism before this eye with base down, which brings the streak through the blaze, measures the amount. On the other hand, if it is above, the other eye tends upward or has hyperphoria, and the prism before the same eye, base up, measures it.

Deviation of either eye above the other of one or more degrees may cause distress.

Thus, this simple instrument may be used by the general practitioner even without prisms, and valuable inferences drawn as to the bearing of the state of the recti eye-muscles on the cause of nervous symptoms. Of course, the greatest light is shed upon the case when this test is made after errors of refraction have been corrected, and in connection with measurements of the deviations, if present, by prisms.

This test may also be extended to the action of the muscles when the eyes are in accommodation, by bringing the test-light to positions near the patient.

This instrument can be obtained of the Fox Optical Co., in this city.

DISCUSSION.

Dr. Grosvenor asked whether it had been demonstrated that an eye when covered would deviate, on vision with the other eye being permitted.

Dr. W. C. Phelps asked what Stevens's idea concerning the relation of eye trouble and nervous symptoms was.

Dr. Falk asked how we were to know whether the patient has estimated the relation of the light and the streak of light correctly.

Dr. A. A. Hubbell, in closing, said that it was a demonstrated fact that the covered eye, on efforts at vision by the other, would deviate. Stevens's idea was, that when the want of equilibrium between the various eye muscles becomes so great that single vision becomes difficult or impossible, then nervous symptoms ensue. He had presented this test because of its simplicity, and because, with prisms, the diplopia is much more difficult for the patient to interpret, than the relation of the lamp and the light streak made by the cylinder.

Dr. E. H. Long then read a paper on Indigestion.

DISCUSSION.

Dr. Dorr believes that the most study concerning indigestion ought to be placed on its relation with microörganisms. The question is a very complicated one, as there are so many modifying factors.

Dr. Burghardt believes that locality and water often exert considerable influence on digestion.

Dr. Grosvenor thought that the mind had much influence on the condition of digestion.

Dr. E. H. Long said, in closing, that the point he wished most particularly to emphasize and bring out, was the use of antiseptics in digestive troubles. He does not believe in the indiscriminate use of pepsin. Lavage is practically a step in antisepsis, and thus is so often of much benefit.

It was announced that the subject for the August meeting would be Hay Fever, Dr. F. W. Hinkel taking its pathology and etiology, and Dr. F. H. Potter its treatment.

